# Telemonitoring in Inflammatory Bowel Disease: Findings from the TIGE-Rus Randomized Controlled Trial

**DOI:** 10.3390/jcm15124800

**Published:** 2026-06-20

**Authors:** Dina A. Akhmedzyanova, Yuliya F. Shumskaya, Kristina V. Charaya, Yuriy A. Vasilev, Anton V. Vladzymyrskyy, Yulya A. Alymova, Ivan A. Blokhin, Roman V. Reshetnikov, Irina V. Kuprina, Olga V. Taschyan, Marta V. Yurazh, Marina G. Mnatsakanyan

**Affiliations:** 1 Research and Practical Clinical Center for Diagnostics and Telemedicine Technologies of the Moscow Health Care Department, Moscow 127051, Russia; 2 The First Sechenov Moscow State Medical University (Sechenov University), Moscow 119991, Russia

**Keywords:** inflammatory bowel disease, ulcerative colitis, Crohn’s disease, mHealth, telemonitoring

## Abstract

**Background:** Telemedicine is increasingly used in inflammatory bowel disease (IBD), but its effects on quality of life (QoL) and psychological outcomes remain unclear. **Objectives**: This study aimed to evaluate the impact of 6-month telemonitoring on QoL, disease activity, treatment adherence, psychological well-being, patient satisfaction, and healthcare utilization. **Methods**: This randomized, open-label, single-center study conducted in Russia (July 2023–December 2024) included adults with ulcerative colitis or Crohn’s disease, who were assigned 1:1 to telemonitoring or standard care. The intervention involved monthly remote assessments and access to a web-based platform containing educational information, disease activity assessment, and a chat with a gastroenterologist. The primary outcome was health-related QoL (SIBDQ). Exploratory outcomes included general QoL (WHOQOL-26), psychological well-being (HADS), alexithymia (TAS-26), visceral sensitivity (VSI), treatment adherence (GMAS), patient satisfaction (PSQ-18), achievement of clinical remission, and healthcare utilization. **Results**: Sixty-eight patients completed the study (32 intervention, 36 control). Telemonitoring was associated with lower anxiety levels (β = −1.76, *p* = 0.021), reduced visceral sensitivity (β = −5.08, *p* = 0.039), and higher medication adherence (β = 1.75, *p* = 0.008). No significant associations were observed for SIBDQ, WHOQOL-26 domains, depressive symptoms, alexithymia, achievement of clinical remission, or patient satisfaction with care (*p* > 0.05). Patients in the telemonitoring group also required fewer outpatient visits (*p* < 0.001), with no difference in hospitalizations. Within-group analysis demonstrated improvements in QoL, treatment adherence, visceral sensitivity, and disease activity in the telemonitoring group, but not in the controls. **Conclusions**: Six-month telemonitoring in IBD was associated with lower anxiety, reduced visceral sensitivity, improved treatment adherence, and fewer outpatient visits. The health-related QoL assessed by the SIBDQ did not differ compared to standard care. No clear clinical disadvantage compared with standard care was detected during the study period.

## 1. Introduction

Inflammatory bowel disease (IBD), comprising ulcerative colitis (UC) and Crohn’s disease (CD), is a group of chronic, relapsing–remitting inflammatory disorders of the gastrointestinal tract that lead to debilitating symptoms, including bloody stools, abdominal pain, and fatigue [1]. IBD substantially impairs general quality of life (QoL) and is associated with a considerable psychological burden, including anxiety, depression, and visceral hypersensitivity that may influence the disease course and perceived symptom severity [2]. Depression and anxiety can reduce treatment adherence in patients with IBD, increase the risk of relapse, and make it harder to follow prescribed therapies [3]. This underscores the importance of monitoring and targeting these components of health status in patients with IBD [4].

Standard care for patients with IBD relies on scheduled outpatient clinic visits [5]. However, a limitation is that routine appointments may not coincide with disease flares. Given the unpredictable nature of IBD, this mismatch can significantly compromise the health-related QoL (HRQoL) [6]. Scheduled visits can lead to significant delays in diagnostic testing and initiation of therapy, reduced treatment adherence, decreased HRQoL and satisfaction with medical care, and inadequate monitoring of complications [7]. Consequently, current guidelines advocate for tighter disease control and early treatment optimization [5,8], which, in turn, requires adaptive approaches to disease monitoring.

Telemedicine, including telemonitoring and remote consultations, is a promising approach for facilitating continuous monitoring and offers patients the opportunity to report symptoms in real time, receive timely medical advice, and access educational resources that enhance disease awareness [9,10]. A growing body of evidence suggests that telemedicine may improve patient satisfaction and strengthen medication adherence [11]. Studies indicate that telemedicine has generally neutral effects on anxiety and depression outcomes in IBD. In a review by Gravina et al. [12], most studies reported no significant effect on anxiety or depression levels, and similar findings were presented in the systematic review by Pang et al. [13]. However, evidence regarding patient-reported outcomes remains mixed. In particular, HRQoL outcomes across telemedicine interventions in IBD are reported as highly heterogeneous, as emphasized in the Cochrane systematic review [14]. Between-study comparability is also limited because telemedicine interventions assess different parameters. Many programs focus on symptoms and healthcare utilization, whereas psychological distress and symptom perception are assessed inconsistently or not at all. When developing the TIGE-Rus (Telemonitoring for IBD Goodness Examination in Russia) intervention and selecting outcomes, we surveyed gastroenterologists to choose a practical set of measures for telemedicine follow-up that reflects common patient problems in routine care. Notably, visceral sensitivity and alexithymia are not routinely assessed in IBD care and, to our knowledge, have not been systematically evaluated as outcomes in telemonitoring trials in IBD. Including these measures provides a more comprehensive assessment of patient status and may help clarify the mechanisms through which telemonitoring could influence patient experience [4].

To date, no randomized controlled trials (RCTs) have evaluated the effectiveness of telemonitoring interventions in Russian patients with IBD. Consequently, the effectiveness of such approaches within the Russian healthcare system remains unclear. Thus, given the importance of monitoring a broad range of parameters, including psychological well-being in IBD patients, the limitations of the existing evidence, and the need to validate previous findings in the Russian population, we initiated the present study. The TIGE-Rus is the first Russian study designed to assess whether a 6-month telemonitoring intervention in adult patients with UC and CD can improve HRQoL, clinical activity, treatment adherence, psychological well-being, satisfaction with medical care, and the frequency of outpatient visits and hospitalizations.

## 2. Materials and Methods

### 2.1. Study Design

The study description is reported in accordance with the CONSORT (Consolidated Standards of Reporting Trials) guidelines [15] (Appendix A). The TIGE-Rus trial was a randomized, open-label, parallel-group, controlled, single-center study conducted from July 2023 to December 2024 to assess whether 6 months of telemonitoring improves outcomes compared with standard in-person care in patients with IBD. The study design has been described previously [16]. This study was registered at ClinicalTrials.gov under the identifier NCT05994716 on 8 August 2023, updated on 5 February 2024 [17]. The study protocol was approved by the Local Ethics Committee (protocol No. 11-23 dated 15 June 2023). Because of the nature of the intervention (telemedicine versus in-person care), blinding of participants and investigators was not feasible [18]. All patients were enrolled after providing written informed consent.

The study included adult patients admitted to the Gastroenterology Department of Sechenov University Hospital, Moscow, Russia.

The inclusion criteria were:

1. Age ≥ 18 years;
2. A confirmed diagnosis of IBD based on standard diagnostic criteria. The diagnosis of UC or CD had been established before study enrollment by treating gastroenterologists based on a combination of clinical, endoscopic, histological, and radiological findings, according to clinical guidelines [19,20].

The non-inclusion criteria were:

1. Malignancy requiring active treatment;
2. Decompensated comorbid conditions severe enough to pose serious health risks or complicate the assessment of trial outcomes;
3. Pregnancy;
4. Participation in other clinical studies;
5. Lack of technical skills or absence of appropriate technology to participate in the telemedicine intervention (e.g., difficulty using a smartphone or computer);
6. Inability to read or understand the informed consent form;
7. Liver cirrhosis;
8. Mental illness or psychiatric disorders that would prevent signing the informed consent form or understanding the potential consequences of participation;
9. Refusal to provide written informed consent.

The exclusion criteria were as follows:

1. Withdrawal of informed consent at any stage of the study;
2. Development of conditions that made further participation unsafe or impossible;
3. Pregnancy during the study period;
4. Participation in another clinical study during the trial period.

The study consisted of three stages:

(1) The first stage involved the enrolment of patients with IBD before discharge from the Gastroenterology Department. Treatment was provided in accordance with current clinical guidelines [19,20]. After providing written informed consent, patients were randomly assigned to either the telemonitoring group or the standard care group (control group). Upon enrolment, each participant was assigned a unique identification number, and all study data were pseudonymized. Patient-identifying information was accessible only to authorized members of the research team, in accordance with the study protocol and regulatory requirements.

After providing informed consent, patients completed the following questionnaires:

- The Short Inflammatory Bowel Disease Questionnaire (SIBDQ), used to assess HRQoL [21];
- The World Health Organization Quality of Life (WHOQOL-26), used to assess general QoL across four domains: physical health, psychological health, social relationships, and environment [22];
- The General Medication Adherence Scale (GMAS) [23];
- The Hospital Anxiety and Depression Scale (HADS) [24];
- The Visceral Sensitivity Index (VSI) [25];
- The Toronto Alexithymia Scale (TAS-26) [26];
- The Patient Satisfaction Questionnaire (PSQ-18) [27];
- The Harvey-Bradshaw Index (HBI), used to assess clinical activity of CD [28];
- The Simple Clinical Colitis Activity Index (SCCAI), used to assess clinical activity of UC [29].

The questionnaires were selected in accordance with the developed methodology [30].

Demographic and clinical characteristics were collected at the baseline visit. Endoscopic activity was assessed during ileocolonoscopy using the Mayo endoscopic subscore (MES) [31] for UC, and the Simple Endoscopic Score (SES-CD) for CD [32], and at least two biopsies were obtained from each of the five colonic segments (right colon, transverse colon, descending colon, sigmoid colon, and rectum) as well as from the terminal ileum for histological assessment. In some patients, IBD activity was also assessed using computed tomography (CT) and/or magnetic resonance enterography (MRE) at hospital admission.

All participants were Russian-speaking, and Russian was used as the language of communication with patients in both groups, including interactions conducted through the platform.

Before initiation of follow-up, patients in the telemonitoring group received an addendum to the informed consent, describing actions to be taken in emergency situations, and contact information for emergency medical services. The addendum has been published elsewhere [16].

(2) The second stage involved follow-up care over the 6-month study period.

Patients in the control group were managed by a physician in the Gastroenterology Department of Sechenov University Hospital and received an in-person consultation with a gastroenterologist, along with follow-up recommendations regarding treatment, post-discharge care, and diet. Patients in the control group received standard post-discharge care in accordance with clinical guidelines [19,20]. Follow-up visits were scheduled based on patients’ clinical needs. Participants in the control group did not have access to the telemonitoring platform, monthly telephone assessments, educational materials delivered through the platform, or direct communication with study gastroenterologists via chat. Any additional consultations or urgent assessments were arranged through the usual healthcare system using standard referral pathways.

Patients in the telemonitoring group were given access to a web-based platform and monitored by the investigators. Follow-up was provided by board-certified gastroenterologists. Through the platform, patients completed monthly clinical activity questionnaires; could contact a gastroenterologist via chat or phone upon request; and received educational information about their condition, psychological well-being, and recommendations on diet. Communication was asynchronous, with responses provided within 24 h. In accordance with the individualized treatment plan prescribed by the physician after discharge, patients could be advised to undergo follow-up laboratory testing. The results of these tests could be uploaded to their personal account on the platform for physician review and assessment.

Psychological support was provided through continuous access to physician communication and educational materials. The educational materials included not only information about the disease and its treatment but also recommendations for psychological well-being. Examples of topics of educational materials are presented in Appendix B. Examples of texts were previously published elsewhere [16].

Safety monitoring was performed throughout the study period. All patients were instructed to immediately seek urgent medical care in the presence of alarm symptoms, including severe abdominal pain, persistent gastrointestinal bleeding, or fever. In the event of deviations in predefined monitored parameters, patients were instructed to notify the healthcare professional responsible for monitoring within two hours. In the event of critical abnormalities, patients were referred for an in-person consultation, clinical assessment, and treatment adjustment within 24 h. The detailed monitoring protocol has been published previously [16].

Investigators continuously assessed patient-reported symptoms during follow-ups to detect potential safety concerns. Investigators also conducted monthly phone surveys of the patients in the telemonitoring group to assess their symptoms and detect signs of disease exacerbation. The surveys included questions on disease activity, medication use, treatment adherence, treatment satisfaction, and HRQoL. The full survey form has been published elsewhere [16]. Appropriate recommendations were provided based on the survey responses. Additional telemedicine consultations were arranged for these patients as needed. Patients requiring further diagnostic evaluation were invited to the outpatient clinic for in-person assessment. Those not requiring hospitalization received treatment, and patients needing close follow-up were re-evaluated. If hospitalization was necessary, patients were admitted directly to the Gastroenterology Department for either planned or emergency care for the following reasons:

- Severe course of IBD and/or the presence of complications;
- Indications for specialized IBD treatment (surgical intervention, corticosteroid and cytostatic therapy, and targeted therapy);
- The need to perform complex interventional diagnostic medical procedures.

The response algorithm is provided in Appendix C.

(3) The third stage involved the evaluation and comparison of the effectiveness of follow-up after six months. Patients completed the questionnaires again (SIBDQ, WHOQOL-26, GMAS, HADS, VSI, TAS-26, PSQ-18, HBI, and SCCAI).

**Figure 1:**
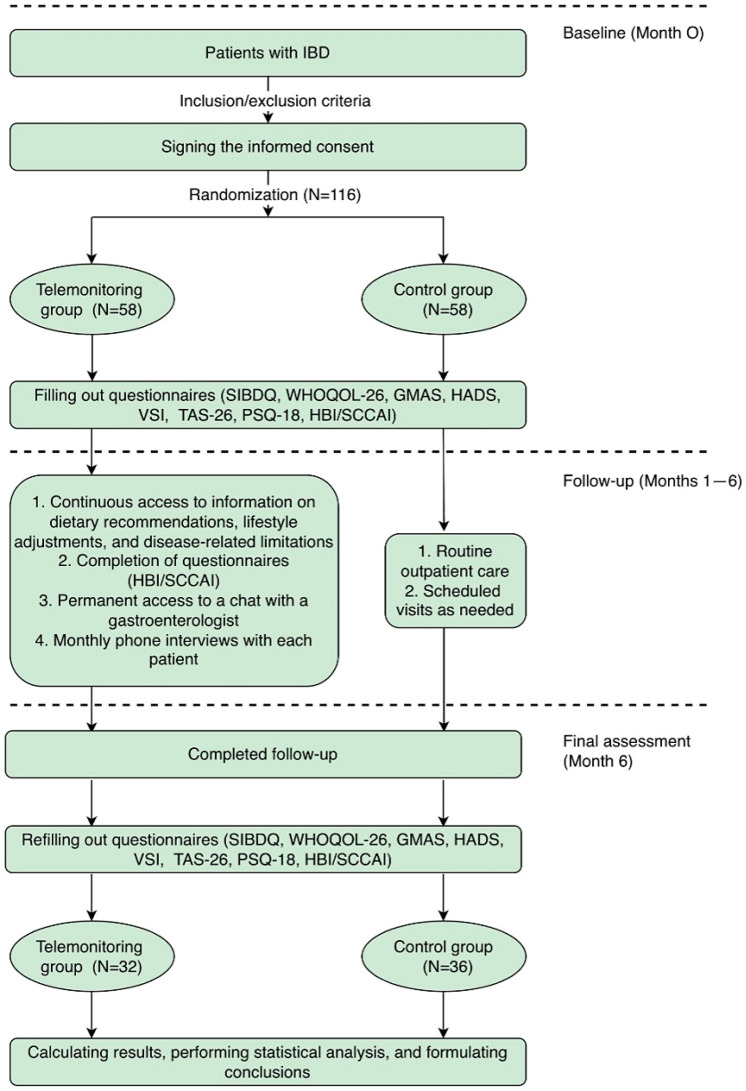
Study design and telemonitoring intervention workflow. GMAS—General Medication Adherence Scale; HADS—Hospital Anxiety and Depression Scale; HBI—Harvey-Bradshaw Index; IBD—Inflammatory bowel disease; PSQ-18—Patient Satisfaction Questionnaire; SCCAI—Simple Clinical Colitis Activity Index; SIBDQ—Short Inflammatory Bowel Disease Questionnaire; TAS-26—Toronto Alexithymia Scale; VSI—Visceral Sensitivity Index; WHOQOL-26—World Health Organization’s QoL.

The study design is shown in Figure 1.

Patients with IBD were enrolled at hospital discharge and randomized in a 1:1 ratio to either standard care (control group) or telemonitoring. Baseline assessments included evaluation of HRQoL, general and psychological well-being, alexithymia, visceral sensitivity, treatment adherence, patient satisfaction, disease activity, and healthcare utilization.

Patients in the telemonitoring group received access to a web-based platform containing educational materials and communication tools, completed monthly clinical activity questionnaires, and could contact healthcare professionals via an online chat. Patients in the control group received standard outpatient follow-up according to routine clinical practice. After 6 months of follow-up, patients in both groups were asked to complete the same questionnaires again. The observation period for each patient was 6 months from the time of inclusion in the study.

### 2.2. Randomization

The randomization sequence was generated by an independent researcher who was not involved in patient recruitment using a computer-generated random number list. Participants were randomly assigned in a 1:1 ratio to either the telemedicine or control group. Group allocation was concealed using sequentially numbered, sealed, opaque envelopes prepared by a researcher who was not involved in the trial. After a participant had provided written informed consent and was enrolled in the study, the next envelope in sequence was opened to reveal the treatment assignment. Investigators responsible for recruitment had no access to the randomization sequence before allocation.

### 2.3. Endpoints

The primary endpoint was the change in HRQoL assessed using the SIBDQ questionnaire.

Exploratory endpoints included:

– Change in general QoL and well-being according to the WHOQOL-26 questionnaire;
– Changes in medication adherence according to the GMAS;
– Changes in psychological well-being according to the HADS;
– Changes in visceral sensitivity according to the VSI;
– Changes in alexithymia according to the TAS-26;
– Changes in satisfaction with medical care according to the PSQ-18;
– Achievement of clinical remission according to the HBI for CD and the SCCAI for UC;
– Changes in the number of in-person visits and hospitalizations.

Additionally, binary outcomes were evaluated to assess the association between telemonitoring and patient-reported endpoints at 6 months. These analyses were considered exploratory. The following dichotomous outcomes were analyzed: optimal HRQoL (SIBDQ ≥ 50), presence of anxiety symptoms (HADS-Anxiety ≥ 8), presence of depression symptoms (HADS-Depression ≥ 8), presence of visceral hypersensitivity (VSI > 10), adequate medication adherence (GMAS ≥ 27), and presence of alexithymia (TAS-26 > 62).

### 2.4. Sample Size

The sample size calculation was based on testing the null hypothesis that there is no difference in the mean change in HRQoL (SIBDQ score) between the telemonitoring and control groups against an alternative hypothesis that a difference exists. The standard deviation and the expected difference in HRQoL between groups were derived from studies of patients with IBD assessed using the SIBDQ. A standard deviation of 12.52 points was adopted according to the study by Sun et al. [33]. The expected difference between the study groups was selected to be smaller than the clinically significant change in HRQoL, as defined by Jowett et al. [34] and taken to be 10 points. Allowing for a potential loss to follow-up and incomplete records, at least 64 patients (32 in the control group and 32 in the intervention group) were required to be included in the study to detect a difference between the groups with 80% statistical power and a two-sided type I error rate of 0.05.

### 2.5. Statistical Analysis

Statistical analyses were conducted using predefined analysis datasets in accordance with the principles outlined in the International Council for Harmonisation of Technical Requirements for Pharmaceuticals for Human Use (ICH) E9 guideline [35]. The full analysis set (FAS) served as the primary analysis population and included all enrolled participants with available outcome data. In the FAS, participants were analyzed according to the group to which they were originally assigned, following the intention-to-treat principle.

The per-protocol (PP) population excluded participants who withdrew from the study before completion of follow-up or who did not complete the SIBDQ questionnaire at any study time point. The study was powered for the primary endpoint (SIBDQ).

Protocol deviations were reviewed during data verification. No major deviations that could substantially affect the interpretation of the primary outcome were identified.

The results were analyzed only after follow-up had been completed for all included patients. Questionnaire scores were calculated according to the scoring guides provided by the questionnaire developers [21,22,23,24,25,26,27,28,29].

Continuous variables were tested for normality using the Shapiro–Wilk test and presented as median and interquartile range (IQR). Categorical variables were presented as percentages. Descriptive statistics were used to characterize the study population and to identify any outliers in the demographic and clinical data. No imputation methods were applied for missing data. Analyses were performed using available data only (complete-case analysis).

Hypothesis testing was conducted for primary and exploratory outcomes. Quantitative variables were compared between groups using the Wilcoxon rank sum test, and qualitative variables were compared using Fisher’s exact test. For within-group comparisons between baseline and 6-month measurements, the Wilcoxon signed-rank test was used, and qualitative variables were compared using McNemar’s test or the Stuart–Maxwell test.

To assess the association between the type of follow-up and outcomes, linear regression analysis adjusted for baseline values of the respective outcome measure was used, with results presented as β coefficients and 95% confidence intervals (CIs). To evaluate the effect of the type of follow-up on the achievement of binary endpoints, logistic regression analysis was used, with results reported as odds ratios (ORs) and 95% CIs.

To control the false discovery rate arising from multiple simultaneous comparisons of study outcomes, the Benjamini–Hochberg (BH) procedure was applied to the set of between-group and within-group outcome analyses.

All analyses were performed using R version 4.2.0. A *p*-value < 0.05 was considered statistically significant.

## 3. Results

### 3.1. Study Population

**Figure 2:**
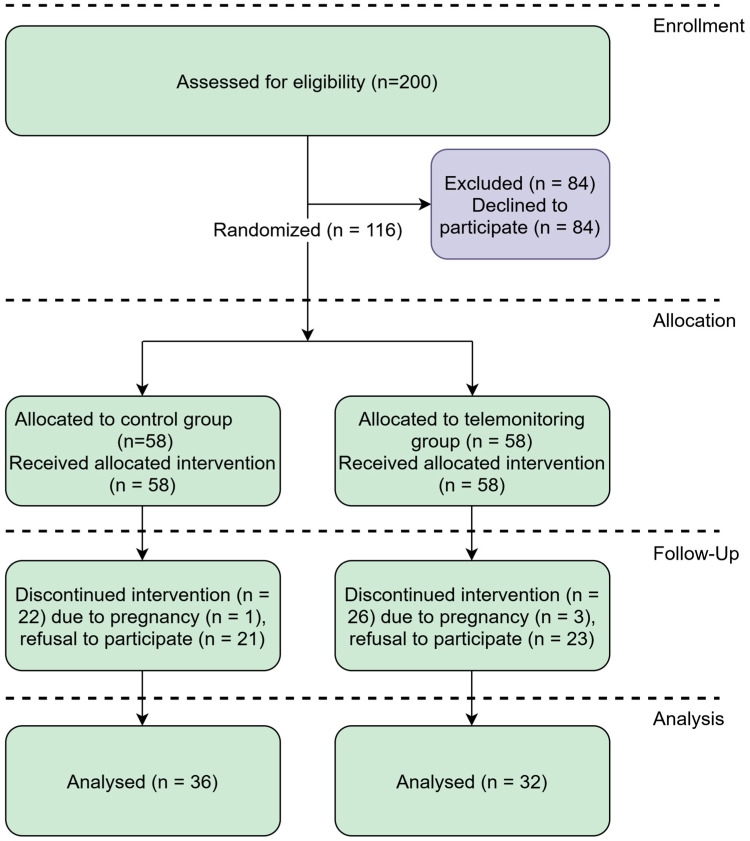
CONSORT flow diagram.

We invited 200 patients with IBD who met the inclusion criteria to participate in the study, of whom 84 declined to provide informed consent (Figure 2). Eligible patients (*n* = 116) provided written informed consent and were randomly assigned to either the telemonitoring group (*n* = 58) or the standard care group (*n* = 58). All included patients completed the questionnaires.

The baseline characteristics of the enrolled patients (FAS) are presented in Appendix D, Table A1.

Subsequently, 26 patients in the telemonitoring group were excluded (3 due to pregnancy and 23 who declined to continue participation). In the control group, 22 patients were excluded (1 due to pregnancy and 21 who declined to continue participation).

The final cohort included 68 patients [36], 38 (55.9%) of whom were men. The median age was 30.5 years (IQR, 25.0–41.0). Thirty-five patients (51.5%) had UC and 33 (48.5%) had CD. In the final analysis, 32 patients (47.1%) were included in the telemonitoring group and 36 (52.9%) to the control group. At baseline, 39 patients (57.4%) were in clinical remission and 29 (42.6%) had active disease, including 23 (33.8%) with mild activity and 6 (8.8%) with moderate activity. The groups were comparable in terms of demographic and clinical characteristics, as shown in Table 1.

No statistically significant differences were observed between groups in baseline QoL, treatment adherence, and psychological characteristics after adjustment for multiple comparisons (Table 2).

**Figure 3:**
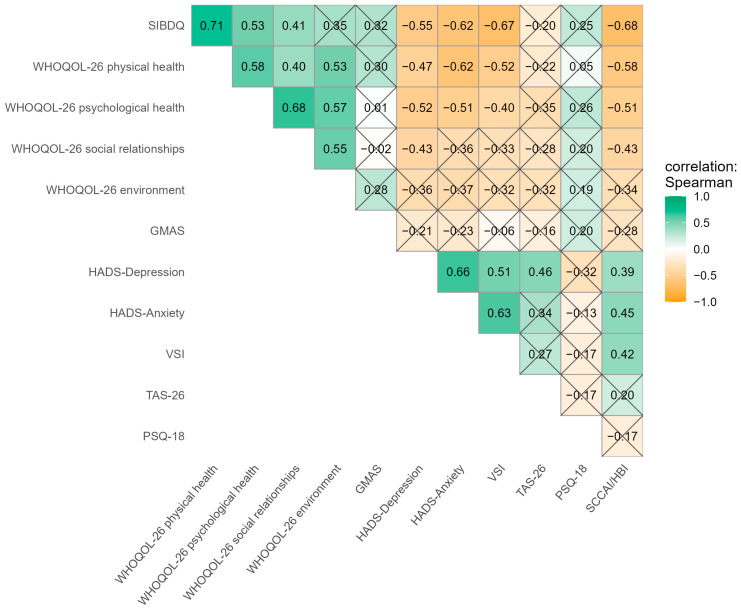
Correlation matrix of baseline patient-state indicators (Spearman rank correlation). The symbol X indicates correlations that were not statistically significant (*p* ≥ 0.05). Disease activity was assessed using HBI/SCCAI. The number of observations varied across individual parameters depending on data availability: clinical activity (SCCAI/HBI), HRQoL (SIBDQ), *n* = 68; general QoL (WHOQOL-26), *n* = 67; anxiety (HADS-Anxiety), depression (HADS-Depression) and visceral sensitivity (VSI), *n* = 66; treatment adherence (GMAS), alexithymia (TAS-26), *n* = 65; treatment satisfaction (PSQ-18), *n* = 53. GMAS—General Medication Adherence Scale; HADS—Hospital Anxiety and Depression Scale; HBI—Harvey–Bradshaw Index; PSQ-18—Patient Satisfaction Questionnaire; SCCAI—Simple Clinical Colitis Activity Index; SIBDQ—Short Inflammatory Bowel Disease Questionnaire; TAS-26—Toronto Alexithymia Scale; VSI—Visceral Sensitivity Index; WHOQOL-26—World Health Organization’s QoL.

Correlation analysis revealed a coherent pattern of associations among disease activity, HRQoL, and psychological distress in patients with IBD (Figure 3).

### 3.2. Outcomes

#### 3.2.1. Between-Group Comparison

Between-group comparisons at 6 months are presented in Table 3. No statistically significant differences were observed between the telemonitoring and control groups for the primary outcome. Specifically, SIBDQ scores did not differ significantly between the groups (*p* = 0.374).

For exploratory outcomes, no significant differences were observed across all parameters: the WHOQOL-26 domains, including physical health (*p* = 0.374), psychological health (*p* = 0.63), social relationships (*p* = 0.874), and environmental health (*p* = 0.671); visceral sensitivity (*p* = 0.366); anxiety (*p* = 0.366); depression (*p* = 0.366); medication adherence (*p* = 0.374); alexithymia (*p* = 0.874); patient satisfaction (*p* = 0.874); or clinical disease activity (*p* = 0.366).

At 6 months, the median number of outpatient consultations with a gastroenterologist was lower in the telemonitoring group than in the control group (3 [1.5; 4] vs. 7 [5; 9]). Hospital readmissions occurred in 31 of 32 patients in the telemonitoring group and 35 of 36 patients in the control group. The median number of hospital admissions due to disease flares did not differ between the groups (1 [1; 1.5] vs. 1 [1; 2]). Patients in the telemonitoring group used the online chat function a median of 6 [3.5; 6] times during follow-up.

#### 3.2.2. Within-Group Dynamics

In the telemonitoring group, over the 6-month period, there was a statistically significant increase in SIBDQ (*p* = 0.004) and GMAS (*p* = 0.004) scores, an improvement in the WHOQOL-26 physical health (*p* = 0.008), and a decrease in VSI score (*p* = 0.004). Furthermore, the number of patients with severe visceral hypersensitivity decreased (*p* = 0.027) (Appendix D, Table A2).

When assessing the changes in the measured indicators in the control group over 6 months, no statistically significant differences were observed (*p* > 0.05; Appendix D, Table A3).

#### 3.2.3. Associations Between Telemonitoring and Patient-Reported Outcomes

**Figure 4:**
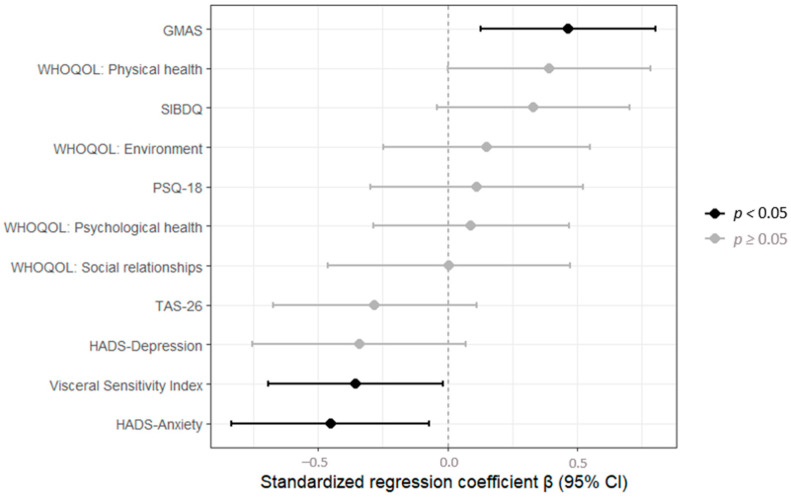
Associations of telemonitoring with patient-reported outcomes after 6 months of follow-up. Forest plot showing standardized regression coefficients (β) and 95% confidence intervals from linear regression models evaluating the association between telemonitoring and patient-reported outcomes after 6 months. Each model was adjusted for the baseline value of the corresponding outcome. Negative coefficients indicate lower values in the telemonitoring group, whereas positive coefficients indicate higher values compared with the standard care group. Black markers represent statistically significant associations (*p* < 0.05), while grey markers represent non-significant associations. GMAS—General Medication Adherence Scale; HADS—Hospital Anxiety and Depression Scale; PSQ-18—Patient Satisfaction Questionnaire; SIBDQ—Short Inflammatory Bowel Disease Questionnaire; TAS-26—Toronto Alexithymia Scale; VSI—Visceral Sensitivity Index; WHOQOL-26—World Health Organization’s QoL.

Associations between telemonitoring and patient-reported outcomes are presented in Figure 4, while non-standardized regression coefficients are provided in Appendix E, Table A4. After adjustment for baseline values, telemonitoring was associated with lower anxiety levels (β = −1.76, 95% CI −3.24 to −0.28; *p* = 0.021), reduced visceral sensitivity (β = −5.08, 95% CI −9.89 to −0.26; *p* = 0.039), and higher medication adherence (β = 1.75, 95% CI 0.48 to 3.02; *p* = 0.008). No significant associations were observed for HRQoL, WHOQOL-26 domains, depression, alexithymia, or patient satisfaction with care (all *p* > 0.05; Appendix E, Table A5).

Logistic regression analysis demonstrated that the use of telemonitoring was associated with a lower likelihood of anxiety symptoms (OR 0.25, CI 0.07–0.90, *p* = 0.034; Appendix E, Table A5).

### 3.3. Subgroup Analysis According to IBD Subtype

A subgroup analysis according to IBD subtype was performed to assess the association between telemonitoring and endpoints achievement. No statistically significant effect of telemonitoring on the outcomes was observed in patients with CD (Table 4). The VSI score tended to be lower in the telemonitoring group (β = −7.04; *p* = 0.051).

In patients with UC, telemonitoring was associated with a significantly higher SIBDQ score (β = 6.46; *p* = 0.035), better WHOQOL-26 physical health domain score (β = 0.085; *p* = 0.034), and greater treatment adherence according to the GMAS (β = 2.17; *p* = 0.029) (Table 5).

## 4. Discussion

In this RCT, 6 months of telemonitoring were associated with improvements in psychological well-being, treatment adherence, and visceral sensitivity, while no clear differences were observed between groups in clinical disease activity scores or hospitalization rates. No clear clinical disadvantage compared with standard care was observed during the 6-month follow-up period.

The primary endpoint of our study was the change in HRQoL among patients with IBD, assessed using the SIBDQ. The WHOQOL-26 domains were evaluated as exploratory outcomes. Improvements in HRQoL were observed in both groups; after 6 months, there were no statistically significant differences in HRQoL scores between the groups (*p* = 0.374). Regression analysis also showed no significant effect of the type of follow-up on changes in QoL, as assessed by both the SIBDQ (HRQoL) and WHOQOL-26 (general QoL). The Cochrane review emphasizes variability in interventions, populations, outcomes, and follow-up, which limits comparability across studies and makes consistent effects on HRQoL difficult to demonstrate [14]. At the same time, the meta-analysis by Pang L. et al. reported that telemonitoring significantly improved HRQoL [13]. Differences between reviews and individual trials may reflect variation in sample size, intervention intensity, follow-up duration, and analytical choices. These discrepancies may be due to methodological differences between systematic reviews, including variations in sample size and analytical approaches. The observed trend toward improved HRQoL in our study may reflect improvements in psychological well-being.

Although the telemonitoring group demonstrated improvements in QoL (HRQoL and physical health in general QoL, Appendix D, Table A2), the absence of a significant effect of the type of follow-up on HRQoL should be interpreted with caution. First, HRQoL instruments may require larger sample sizes to detect meaningful changes, particularly when baseline scores are relatively high. Second, improvements in psychological parameters often precede measurable changes in broader HRQoL indices. Third, the trial duration may have been insufficient to capture downstream effects on HRQoL resulting from improved adherence or reduced psychological distress. Taken together, these considerations suggest that the neutral HRQoL findings do not diminish the clinical relevance of the psychological and behavioral effects observed.

The baseline correlation matrix (Figure 3) shows that poorer HRQoL in patients with IBD is associated not only with disease activity but also with broader psychological distress, including anxiety and depression, as well as symptom perception, reflected by visceral hypersensitivity. These findings are consistent with previous studies [37]. Therefore, improvements in these parameters may theoretically contribute to enhanced HRQoL in patients with IBD.

In our study, telemonitoring was associated with a significant reduction in anxiety levels (β = −1.76, 95% CI −3.24 to −0.28; *p* = 0.021). In contrast, no significant effect on depression was observed (Appendix E, Table A4). Psychological comorbidity is highly prevalent in IBD and is associated with increased symptom perception, a higher risk of relapse, reduced treatment adherence, and greater healthcare utilization [38,39]. Sweeney L. et al., in a systematic review, demonstrated that higher levels of anxiety and depression are associated with greater pain intensity in IBD [40]. This may be explained by the relationship between anxiety, depression, and visceral hypersensitivity, which contributes to hyperalgesia. A systematic review by Eugenicos M.P. et al. demonstrated that symptoms of anxiety and depression exacerbate the clinical course of IBD, shorten remission periods, and reduce adherence to therapy [38]. Patients with anxiety and depression tend to seek medical care either inappropriately often or only in critical situations requiring urgent intervention, both of which increase healthcare utilization and associated costs [38]. According to a systematic review by Neuendorf R. et al., these alterations could persist even during disease remission [41]. In our study, 19.6% of patients in remission exhibited signs of depression, and 29.4% had signs of anxiety, which is consistent with the published data.

Previous RCTs of telemedicine in IBD have generally demonstrated neutral effects on anxiety and depression [12]. Similar findings are reported in a systematic review by Pang L. et al. [13]. Several mechanisms may explain the significant reduction in anxiety observed in our study. First, our telemonitoring model incorporated structured monthly follow-ups, rapid clinician feedback, and continuous access to disease-specific educational materials. The discrepancies between studies may reflect differences in sample sizes, the use of various psychological assessment tools, and differing follow-up durations. Improving psychological well-being, alongside achieving disease remission, is increasingly recognized as an important treatment goal in IBD [42]. However, this outcome was an exploratory endpoint in the present study, and further research is needed to confirm these findings.

A key novel finding of our study is that telemonitoring was associated with reduced visceral sensitivity (β = −5.08, 95% CI −9.89 to −0.26; *p* = 0.039). To our knowledge, no previous telemedicine study in IBD has included visceral sensitivity as an outcome. While visceral hypersensitivity is a well-established mediator of abdominal pain in functional gastrointestinal disorders, it is increasingly recognized in IBD, even during clinical remission [43]. One possible explanation is that the reduction in visceral hypersensitivity observed in our study may be related to the same mechanisms underlying the decrease in general anxiety, namely, regular interaction with a physician and the resulting increased sense of support. We also cannot exclude the possibility that reduced anxiety contributed directly to the observed reduction in visceral hypersensitivity. The integration of validated instruments for assessing anxiety, depression, and visceral sensitivity provides a detailed psychological profile of IBD patients and allows for a more granular understanding of how telemedicine may influence their disease experience.

Alexithymia is another factor that can contribute to reduced QoL and difficulties in describing and interpreting symptoms in IBD [43]. The lack of an effect of telemonitoring on alexithymia was expected and is consistent with previous studies. This pattern is consistent with alexithymia being a relatively stable trait and may therefore be less likely to change over a short follow-up period.

Changes in patients’ psychological state may result from receiving reliable, comprehensible information about the disease available at the web-based platform, as well as from the sense that they are not facing the challenges of the illness alone. As shown in previous studies, patient education programs for individuals with IBD can improve psychological well-being by reducing health-related concerns, alleviating fear of disease progression, and promoting self-management skills, including strategies for coping with anxiety [44]. In addition, patient education has been shown to play an important role in improving adherence to pharmacological therapy among patients with IBD [45].

Timely access to physicians may have provided emotional support, which contributed to improvements in their psychological well-being. In a meta-analysis by Zolnierek et al., continuous communication with healthcare providers was shown to improve patient adherence to therapy [46], which is particularly important for patients with IBD, as poor adherence is associated with worse outcomes [47]. It has also been demonstrated that the use of electronic communication tools improves the effectiveness of self-management, reduces the number of hospital visits, decreases anxiety levels, and enhances HRQoL [45]. Importantly, the implementation of telemedicine minimized the need for in-person consultations with a gastroenterologist, which may have contributed to improved psychological comfort among patients.

IBD treatment requires long-term, continuous medication use, making strict adherence to medical recommendations essential. Thus, strategies aimed at improving treatment adherence become particularly important [45]. Our findings support a positive effect of telemonitoring on treatment adherence (β = 1.75, 95% CI 0.48 to 3.02; *p* = 0.008), consistent with previous international RCTs [48,49]. It should be noted that assessing the clinical effect of improved adherence is challenging, as 57.4% of patients were in disease remission. Moreover, baseline adherence in our sample was already high: 29.5 GMAS points in the control group and 29 points in the telemonitoring group (GMAS scores above 27 indicate good treatment adherence, while scores above 30 reflect high adherence) [50]. Regression analysis showed that the transition of a patient from the non-adherent group to the adherent group was not associated with the type of follow-up. This may be because treatment adherence was relatively high in our cohort and therefore had limited variability and because adherence is influenced by many factors beyond mood (socioeconomic status and social support, belonging to an ethnic minority, age) [51].

The type of follow-up was not associated with the achievement of clinical remission (OR 3.57, 95% CI 0.99–12.91, *p* = 0.053). This finding is consistent with the results of systematic reviews by Pang L. et al. [13] and Kuriakose Kuzhiyanjal A.J. et al. [52], who also reported no differences in disease activity or remission rates between patients managed in person and via telemedicine. In our study, IBD clinical activity was assessed at the time of hospital discharge, when most patients’ symptoms had already resolved. No significant changes were observed in clinical activity indices over follow-up. Other measures of disease activity were outside the scope of the present study.

The absence of statistically significant differences in clinical activity, hospitalizations, or objective inflammatory measures should not be interpreted as evidence of equivalence between telemonitoring and standard care. The study was powered to detect differences in HRQoL rather than clinical outcomes and was not designed as a noninferiority trial. Therefore, our findings indicate that no clear clinical disadvantage of telemonitoring was detected during the 6-month follow-up period, but larger studies specifically designed to assess clinical outcomes are required before conclusions regarding equivalence or noninferiority can be drawn. It should also be noted that the lack of statistically significant effects observed for binary outcomes, except for anxiety, may be attributable to information loss associated with dichotomization of continuous scales, as well as the limited statistical power of the study sample.

Satisfaction with the quality of healthcare is a key component of a patient-centered approach, particularly in chronic conditions such as IBD [53]. In our study, no significant differences in satisfaction were observed between the control and telemonitoring groups (both groups scored >75 points on the PSQ-18 questionnaire), which is consistent with previously published data [54,55]. These findings suggest that telemedicine may serve as a feasible alternative to traditional models of care while maintaining high levels of patient satisfaction.

The interpretation of our findings should consider not only the reduction in outpatient visits in the telemonitoring group but also the total number of patient–physician contacts. When both remote and face-to-face consultations were taken into account, the overall number of consultations was higher in the telemonitoring group than in the standard care group. This observation supports the argument that patients with IBD have a substantial need for timely medical consultations, which is not always met within the framework of scheduled outpatient visits. Thus, telemedicine provided rapid access to medical advice in response to symptom changes, enabling more frequent interaction with healthcare professionals without increasing the burden on outpatient services.

Consistent with previous RCTs [13], telemonitoring did not statistically significantly reduce hospitalization rates in our study. This was expected given the predominance of patients in remission at enrolment and the short follow-up period. However, remotely monitored patients required fewer outpatient visits. Thus, telemonitoring of patients with IBD could reduce the number of in-person visits without increasing hospitalizations.

The exploratory subgroup analysis suggested that the associations between telemonitoring and patient-reported outcomes may differ according to IBD subtype. In patients with UC, telemonitoring was associated with higher disease-specific quality of life, better physical quality of life, and greater medication adherence, whereas in patients with CD, no statistically significant associations were observed. Although these findings should be interpreted cautiously because of the limited sample size, they may indicate differential responsiveness to telemonitoring interventions across IBD subtypes. Previous studies have demonstrated distinct psychological profiles in patients with UC and CD. In particular, patients with UC have been reported to exhibit higher levels of fears and emotional dysregulation-related traits than patients with CD [56]. These differences may contribute to a greater impact of interventions providing regular feedback, education, and communication with healthcare professionals. However, given the exploratory nature of this analysis, further studies are needed to determine whether the effectiveness of telemonitoring truly differs between UC and CD.

### Limitations

This study has several limitations. First, it was conducted at a single center and included a relatively small number of patients, which may limit the generalizability of the findings. However, although recruitment was performed at one center, participants were referred from multiple regions of Russia, and the final sample met the prespecified minimum sample size, as described in Section 2.4 (“Sample Size”).

Second, the 6-month follow-up period limits conclusions regarding the long-term sustainability of the observed psychological and patient-reported improvements. In addition, a substantial proportion of participants withdrew during follow-up. Although attrition was numerically similar between the study groups and the minimum required sample size was maintained, the high withdrawal rate should be considered when interpreting the results.

Third, the web-based format of the intervention may have influenced participant selection, as it required access to internet-enabled devices and sufficient digital literacy, potentially affecting both the size and demographic composition of the study population. Additionally, because the telemonitoring intervention incorporated more frequent patient–physician contact and educational support than standard care, the study was not designed to distinguish the effects of the digital platform itself from those of increased clinical interaction and reassurance.

Fourth, the assessment of objective inflammatory activity was incomplete. Histological and endoscopic activities at baseline were assessed in 54 (79.4%) and 56 (82.4%) patients, respectively, and not all participants underwent repeated endoscopic or histological evaluation during follow-up. Moreover, fecal calprotectin, C-reactive protein, corticosteroid exposure, treatment escalation, rescue therapy, and modifications of biologic treatment were not prospectively collected as study outcomes. Consequently, this study cannot determine whether telemonitoring influenced disease control or treatment-related outcomes.

Fifth, the subgroup analyses stratified by IBD subtype were exploratory and may have been underpowered because of the limited number of patients in each subgroup. Finally, the study population consisted primarily of patients in remission or with mild IBD; therefore, extrapolation of the findings to patients with more severe disease requires further investigation.

## 5. Conclusions

In this RCT, telemonitoring did not result in a statistically significant improvement in HRQoL assessed by the SIBDQ, compared with standard care. Nevertheless, telemonitoring was associated with improvements in several exploratory outcomes, including anxiety, visceral sensitivity, and treatment adherence. No clear clinical disadvantage compared with standard care was detected during the 6-month follow-up period. Given the limitations of the existing evidence and the variability of prior interventions, our results provide data from a Russian cohort and contribute to validating earlier findings in a different healthcare context and cultural setting. Future large-scale, multicenter studies with longer follow-up are warranted to determine whether these improvements translate into long-term disease outcomes.

## Figures and Tables

**Table 1:** Baseline demographic and clinical characteristics for each group (per-protocol population).

Parameter	Overall ^1^	Control Group ^1^	Telemonitoring Group ^1^	*p*-Value ^2^	BH-Adjusted *p*-Value ^3^
Sex				0.087	0.366
Male	38 (55.9%)	24 (66.7%)	14 (43.8%)		
Female	30 (44.1%)	12 (33.3%)	18 (56.3%)		
Age, years	30.5 [25.0; 41.0]	35.5 [26.0; 41.5]	29.0 [24.0; 38.5]	0.192	0.475
Type of IBD				0.478	0.772
UC	35 (51.5%)	17 (47.2%)	18 (56.3%)		
CD	33 (48.5%)	19 (52.8%)	14 (43.8%)		
Clinical activity (HBI/SCCAI)				0.871	0.915
Remission	39 (57.4%)	20 (55.6%)	19 (59.4%)		
Mild activity	23 (33.8%)	12 (33.3%)	11 (34.4%)		
Moderate activity	6 (8.8%)	4 (11.1%)	2 (6.3%)		
Severe activity	0 (0.0%)	0 (0.0%)	0 (0.0%)		
Clinical activity (HBI/SCCAI), binary				0.809	0.874
Remission	39 (57.4%)	20 (55.6%)	19 (59.4%)		
Flare	29 (42.6%)	16 (44.4%)	13 (40.6%)		
Endoscopic disease activity				0.956	0.956
Remission	18 (31.6%)	9 (29.0%)	9 (34.6%)		
Mild activity	21 (36.8%)	12 (38.7%)	9 (34.6%)		
Moderate activity	14 (24.6%)	8 (25.8%)	6 (23.1%)		
Severe activity	4 (7.0%)	2 (6.5%)	2 (7.7%)		
Missing data	11	5	6		
Histological disease activity				0.223	0.563
No activity	15 (27.8%)	6 (20.0%)	9 (37.5%)		
Activity present	39 (72.2%)	24 (80.0%)	15 (62.5%)		
Missing data	14	6	8		
CT/MRE activity				0.190	0.563
No activity	9 (34.6%)	8 (44.4%)	1 (12.5%)		
Activity present	17 (65.4%)	10 (55.6%)	7 (87.5%)		
Missing data	42	18	24		

^1^ *n* (%); Median [Q1; Q3]. ^2^ Fisher’s exact test; Wilcoxon rank-sum test. ^3^ Benjamini & Hochberg correction for multiple testing. CD—Crohn’s disease; CT—computed tomography; HBI—Harvey–Bradshaw Index; MRE—magnetic resonance enterography; SCCAI—Simple Clinical Colitis Activity Index; UC—ulcerative colitis.

**Table 2:** Baseline QoL, treatment adherence, psychological characteristics and satisfaction with care for each group (per-protocol population).

Parameter	Overall ^1^	Control Group ^1^	Telemonitoring Group ^1^	*p*-Value ^2^	BH-Adjusted *p*-Value ^3^
SIBDQ, score	51.5 [41.0; 59.5]	51.5 [39.5; 59.5]	53.0 [41.0; 59.5]	0.796	0.874
HRQoL (SIBDQ)				0.811	0.874
Suboptimal HRQoL	31 (45.6%)	17 (47.2%)	14 (43.8%)		
Optimal HRQoL	37 (54.4%)	19 (52.8%)	18 (56.3%)		
WHOQOL-26, physical health	0.6 [0.5; 0.7]	0.6 [0.5; 0.7]	0.7 [0.5; 0.8]	0.685	0.874
Missing data	1	0	1		
WHOQOL-26, psychological health	0.7 [0.6; 0.8]	0.7 [0.6; 0.7]	0.7 [0.6; 0.8]	0.086	0.366
Missing data	1	0	1		
WHOQOL-26, social relationships	0.8 [0.6; 0.8]	0.7 [0.5; 0.8]	0.8 [0.7; 0.8]	0.045	0.366
Missing data	1	0	1		
WHOQOL-26, environmental health	0.7 [0.6; 0.8]	0.7 [0.6; 0.8]	0.7 [0.6; 0.8]	0.291	0.612
Missing data	1	0	1		
GMAS, score	29.0 [26.0; 32.0]	29.5 [28.0; 32.0]	29.0 [25.0; 32.0]	0.384	0.671
Missing data	3	0	3		
Medication adherence (GMAS)				0.162	0.426
Non-adherent	18 (27.7%)	7 (19.4%)	11 (37.9%)		
Adherent	47 (72.3%)	29 (80.6%)	18 (62.1%)		
Missing data	3	0	3		
HADS-Depression, score	5.0 [3.0; 8.0]	5.0 [3.0; 9.0]	5.0 [3.0; 7.0]	0.796	0.874
Missing data	2	1	1		
Depression (HADS)				0.103	0.366
No depression	50 (75.8%)	23 (65.7%)	27 (87.1%)		
Subclinical depression	9 (13.6%)	6 (17.1%)	3 (9.7%)		
Clinically significant depression	7 (10.6%)	6 (17.1%)	1 (3.2%)		
Missing data	2	1	1		
HADS-Anxiety, score	7.0 [4.0; 10.0]	7.0 [4.0; 10.0]	7.0 [2.0; 10.0]	0.611	0.874
Missing data	2	1	1		
Anxiety (HADS)				0.783	0.874
No anxiety	38 (57.6%)	19 (54.3%)	19 (61.3%)		
Subclinical anxiety	18 (27.3%)	11 (31.4%)	7 (22.6%)		
Clinically significant anxiety	10 (15.2%)	5 (14.3%)	5 (16.1%)		
Missing data	2	1	1		
VSI, score	32.0 [22.0; 41.0]	33.0 [22.0; 44.0]	29.0 [21.0; 40.0]	0.4	0.671
Missing data	2	1	1		
Visceral sensitivity (VSI)				0.388	0.671
No visceral hypersensitivity	5 (7.6%)	2 (5.7%)	3 (9.7%)		
Moderate visceral hypersensitivity	27 (40.9%)	12 (34.3%)	15 (48.4%)		
Severe visceral hypersensitivity	34 (51.5%)	21 (60.0%)	13 (41.9%)		
Missing data	2	1	1		
TAS-26, score	66.0 [57.0; 70.0]	62.5 [56.0; 69.0]	68.0 [64.0; 71.0]	0.078	0.366
Missing data	3	2	1		
Alexithymia (TAS-26)				0.234	0.52
No alexithymia	26 (40.0%)	17 (50.0%)	9 (29.0%)		
Possible alexithymia	33 (50.8%)	15 (44.1%)	18 (58.1%)		
Clinically significant alexithymia	6 (9.2%)	2 (5.9%)	4 (12.9%)		
Missing data	3	2	1		
PSQ-18, score	78.0 [70.0; 84.0]	78.0 [72.0; 87.0]	78.0 [68.0; 84.0]	0.575	0.874
Missing data	15	14	1		

^1^ *n* (%); Median [Q1; Q3]. ^2^ Fisher’s exact test; Wilcoxon rank-sum test. ^3^ Benjamini & Hochberg correction for multiple testing. GMAS—General Medication Adherence Scale; HADS—Hospital Anxiety and Depression Scale; HRQoL—Health-related quality of life; PSQ-18—Patient Satisfaction Questionnaire; SIBDQ—Short Inflammatory Bowel Disease Questionnaire; TAS-26—Toronto Alexithymia Scale; VSI—Visceral Sensitivity Index; WHOQOL-26—World Health Organization’s QoL.

**Table 3:** Characteristics of the two groups 6 months after study inclusion.

Parameter	Control Group ^1^	Telemonitoring Group ^1^	Difference	*p*-Value ^2^	BH-Adjusted *p*-Value ^3^
Demographic and clinical characteristics					
Clinical activity (HBI/SCCAI)			n/a	0.101	0.366
Remission	24 (66.7%)	28 (87.5%)			
Mild activity	10 (27.8%)	4 (12.5%)			
Moderate activity	2 (5.6%)	0 (0.0%)			
Severe activity	0 (0.0%)	0 (0.0%)			
Clinical activity (HBI/SCCAI), binary			n/a	0.051	0.366
Remission	24 (66.7%)	28 (87.5%)			
Flare	12 (33.3%)	4 (12.5%)			
Patient-reported outcomes					
SIBDQ, score	54.5 [45.5; 59.0]	57.0 [45.0; 63.5]	−4	0.127	0.374
HRQoL (SIBDQ)			n/a	0.999	0.999
Suboptimal HRQoL	12 (33.3%)	10 (31.3%)			
Optimal HRQoL	24 (66.7%)	22 (68.8%)			
WHOQOL-26, physical health	0.6 [0.6; 0.8]	0.7 [0.6; 0.8]	−0.06	0.134	0.374
Missing data	3	0			
WHOQOL-26, psychological health	0.6 [0.6; 0.8]	0.8 [0.6; 0.8]	−0.04	0.315	0.63
Missing data	3	0			
WHOQOL-26, social relationships	0.8 [0.6; 0.8]	0.7 [0.6; 0.8]	0	0.684	0.874
Missing data	3	0			
WHOQOL-26, environmental health	0.7 [0.6; 0.8]	0.8 [0.6; 0.8]	−0.03	0.37	0.671
Missing data	3	0			
GMAS, score	31.0 [29.0; 32.0]	32.0 [30.0; 32.5]	−1	0.129	0.374
Missing data	1	0			
Medication adherence (GMAS)			n/a	0.739	0.874
Non-adherent	6 (17.1%)	4 (12.9%)			
Adherent	29 (82.9%)	27 (87.1%)			
Missing data	1	1			
HADS-Depression, score	6.0 [4.0; 8.0]	4.0 [2.0; 6.0]	2	0.074	0.366
Missing data	2	0			
Depression (HADS)			n/a	0.235	0.52
No depression	22 (64.7%)	26 (81.3%)			
Subclinical depression	11 (32.4%)	5 (15.6%)			
Clinically significant depression	1 (2.9%)	1 (3.1%)			
Missing data	2	0			
HADS-Anxiety, score	8.0 [4.0; 11.0]	5.5 [3.5; 8.5]	2	0.049	0.366
Missing data	3	0			
Anxiety (HADS)			n/a	0.105	0.366
No anxiety	15 (44.1%)	22 (68.8%)			
Subclinical anxiety	9 (26.5%)	6 (18.8%)			
Clinically significant anxiety	10 (29.4%)	4 (12.5%)			
Missing data	2	0			
VSI, score	30.0 [23.0; 41.0]	21.5 [15.5; 28.0]	7	0.025	0.366
Missing data	2	0			
Visceral sensitivity (VSI)			n/a	0.025	0.366
No visceral hypersensitivity	2 (5.9%)	5 (15.6%)			
Moderate visceral hypersensitivity	15 (44.1%)	21 (65.6%)			
Severe visceral hypersensitivity	17 (50.0%)	6 (18.8%)			
Missing data	2	0			
TAS-26, score	63.0 [56.0; 74.0]	63.0 [55.0; 71.5]	−1	0.626	0.874
Missing data	2	0			
Alexithymia (TAS-26)			n/a	0.902	0.924
No alexithymia	16 (47.1%)	15 (46.9%)			
Possible alexithymia	10 (29.4%)	11 (34.4%)			
Clinically significant alexithymia	8 (23.5%)	6 (18.8%)			
Missing data	2	0			
PSQ-18, score	76.5 [69.0; 80.0]	76.0 [67.5; 84.5]	−1	0.768	0.874
Missing data	2	0			

^1^ *n* (%); Median [Q1; Q3]. ^2^ Fisher’s exact test; Wilcoxon rank sum test. ^3^ Benjamini & Hochberg correction for multiple testing. GMAS—General Medication Adherence Scale; HADS—Hospital Anxiety and Depression Scale; HBI—Harvey–Bradshaw Index; HRQoL—Health-related quality of life; PSQ-18—Patient Satisfaction Questionnaire; SCCAI—Simple Clinical Colitis Activity Index; SIBDQ—Short Inflammatory Bowel Disease Questionnaire; TAS-26—Toronto Alexithymia Scale; VSI—Visceral Sensitivity Index; WHOQOL-26—World Health Organization’s QoL.

**Table 4:** Association between telemonitoring and 6-month outcomes in patients with CD.

Parameter	β	95% CI	*p*-Value
SIBDQ	1.09	−3.53–5.70	0.634
WHOQOL-26, physical health	0.015	−0.073–0.104	0.727
WHOQOL-26, psychological health	−0.011	−0.122–0.101	0.847
WHOQOL-26, social relationships	−0.076	−0.259–0.107	0.401
WHOQOL-26, environmental health	0.008	−0.100–0.115	0.887
GMAS	1.15	−0.65–2.95	0.202
HADS Depression	−1.36	−3.32–0.59	0.165
HADS Anxiety	−2.17	−4.77–0.44	0.100
VSI	−7.04	−14.1–0.02	0.051
TAS-26	−2.41	−8.66–3.84	0.436
PSQ-18	3.22	−3.72–10.2	0.348

CI—confidence interval; GMAS—General Medication Adherence Scale; HADS—Hospital Anxiety and Depression Scale; PSQ-18—Patient Satisfaction Questionnaire; SIBDQ—Short Inflammatory Bowel Disease Questionnaire; TAS-26—Toronto Alexithymia Scale; VSI—Visceral Sensitivity Index; WHOQOL-26—World Health Organization’s QoL.

**Table 5:** Association between telemonitoring and 6-month outcomes in patients with UC.

Parameter	β	95% CI	*p*-Value
SIBDQ	6.46	0.48–12.5	0.035 *
WHOQOL-26, physical health	0.085	0.007–0.164	0.034 *
WHOQOL-26, psychological health	0.039	−0.037–0.115	0.307
WHOQOL-26, social relationships	0.061	−0.043–0.166	0.241
WHOQOL-26, environmental health	0.034	−0.033–0.102	0.306
GMAS	2.17	0.24–4.09	0.029 *
HADS-Depression	−0.67	−2.40–1.07	0.440
HADS-Anxiety	−1.53	−3.27–0.20	0.081
VSI	−3.90	−9.46–1.66	0.162
TAS-26	−3.86	−10.4–2.69	0.238
PSQ-18	−2.02	−7.90–3.85	0.482

* *p* < 0.05. CI—confidence interval; GMAS—General Medication Adherence Scale; HADS—Hospital Anxiety and Depression Scale; PSQ-18—Patient Satisfaction Questionnaire; SIBDQ—Short Inflammatory Bowel Disease Questionnaire; TAS-26—Toronto Alexithymia Scale; VSI—Visceral Sensitivity Index; WHOQOL-26—World Health Organization’s QoL.

## Data Availability

The datasets utilized and examined in this study can be obtained from the principal investigator (Akhmedzyanova Dina) upon reasonable request.

## Abbreviations

The following abbreviations are used in this manuscript:

CD	Crohn’s disease
CT	Computed tomography
GMAS	General Medication Adherence Scale
HADS	Hospital Anxiety and Depression Scale
HBI	Harvey–Bradshaw Index
HRQoL	Health-related quality of life
IBD	Inflammatory bowel disease
IQR	Interquartile range
MRE	Magnetic resonance enterography
PSQ-18	Patient Satisfaction Questionnaire-18
QoL	Quality of life
RCT	Randomized controlled trial
SCCAI	Simple Clinical Colitis Activity Index
SIBDQ	Short Inflammatory Bowel Disease Questionnaire
TAS-26	Toronto Alexithymia Scale
UC	Ulcerative colitis
VSI	Visceral Sensitivity Index
WHOQOL-26	World Health Organization Quality of Life Questionnaire

## Appendix A. CONSORT 2025 Checklist

**Section/Topic**	**No**	**CONSORT 2025 Checklist Item Description**	**Reported on Page No.**
Title and abstract			
Title and structured abstract	1a	Identification as a randomised trial	1
	1b	Structured summary of the trial design, methods, results, and conclusions	1
Open science			
Trial registration	2	Name of trial registry, identifying number (with URL) and date of registration	3
Protocol and statistical analysis plan	3	Where the trial protocol and statistical analysis plan can be accessed	3
Data sharing	4	Where and how the individual de-identified participant data (including data dictionary), statistical code and any other materials can be accessed	21
Funding and conflicts of interest	5a	Sources of funding and other support (e.g., supply of drugs), and role of funders in the design, conduct, analysis and reporting of the trial	21
	5b	Financial and other conflicts of interest of the manuscript authors	21
Introduction			
Background and rationale	6	Scientific background and rationale	2
Objectives	7	Specific objectives related to benefits and harms	2
Methods			
Patient and public involvement	8	Details of patient or public involvement in the design, conduct and reporting of the trial	3
Trial design	9	Description of trial design including type of trial (e.g., parallel group, crossover), allocation ratio, and framework (e.g., superiority, equivalence, non-inferiority, exploratory)	3–7
Changes to trial protocol	10	Important changes to the trial after it commenced including any outcomes or analyses that were not prespecified, with reason	N/A
Trial setting	11	Settings (e.g., community, hospital) and locations (e.g., countries, sites) where the trial was conducted	3
Eligibility criteria	12a	Eligibility criteria for participants	3
	12b	If applicable, eligibility criteria for sites and for individuals delivering the interventions (e.g., surgeons, physiotherapists)	N/A
Intervention and comparator	13	Intervention and comparator with sufficient details to allow replication. If relevant, where additional materials describing the intervention and comparator (e.g., intervention manual) can be accessed	N/A
Outcomes	14	Prespecified primary and secondary outcomes, including the specific measurement variable (e.g., systolic blood pressure), analysis metric (e.g., change from baseline, final value, time to event), method of aggregation (e.g., median, proportion), and time point for each outcome	7
Harms	15	How harms were defined and assessed (e.g., systematically, non-systematically)	N/A
Sample size	16a	How sample size was determined, including all assumptions supporting the sample size calculation	7
	16b	Explanation of any interim analyses and stopping guidelines	N/A
Randomisation:			
Sequence generation	17a	Who generated the random allocation sequence and the method used	7
	17b	Type of randomisation and details of any restriction (e.g., stratification, blocking and block size)	7
Allocation concealment mechanism	18	Mechanism used to implement the random allocation sequence (e.g., central computer/telephone; sequentially numbered, opaque, sealed containers), describing any steps to conceal the sequence until interventions were assigned	7
Implementation	19	Whether the personnel who enrolled and those who assigned participants to the interventions had access to the random allocation sequence	7
Blinding	20a	Who was blinded after assignment to interventions (e.g., participants, care providers, outcome assessors, data analysts)	N/A
	20b	If blinded, how blinding was achieved and description of the similarity of interventions	N/A
Statistical methods	21a	Statistical methods used to compare groups for primary and secondary outcomes, including harms	8
	21b	Definition of who is included in each analysis (e.g., all randomised participants), and in which group	7
	21c	How missing data were handled in the analysis	8
	21d	Methods for any additional analyses (e.g., subgroup and sensitivity analyses), distinguishing prespecified from post hoc	8
Results			
Participant flow, including flow diagram	22a	For each group, the numbers of participants who were randomly assigned, received intended intervention, and were analysed for the primary outcome	8–9
	22b	For each group, losses and exclusions after randomisation, together with reasons	8
Recruitment	23a	Dates defining the periods of recruitment and follow-up for outcomes of benefits and harms	3
	23b	If relevant, why the trial ended or was stopped	N/A
Intervention and comparator delivery	24a	Intervention and comparator as they were actually administered (e.g., where appropriate, who delivered the intervention/comparator, how participants adhered, whether they were delivered as intended (fidelity))	4–5
	24b	Concomitant care received during the trial for each group	N/A
Baseline data	25	A table showing baseline demographic and clinical characteristics for each group	9–11
Numbers analysed, outcomes and estimation	26	For each primary and secondary outcome, by group: - the number of participants included in the analysis - the number of participants with available data at the outcome time point - result for each group, and the estimated effect size and its precision (such as 95% confidence interval) - for binary outcomes, presentation of both absolute and relative effect size	13–14
Harms	27	All harms or unintended events in each group	N/A
Ancillary analyses	28	Any other analyses performed, including subgroup and sensitivity analyses, distinguishing pre-specified from post hoc	14–16
Discussion			
Interpretation	29	Interpretation consistent with results, balancing benefits and harms, and considering other relevant evidence	17–20
Limitations	30	Trial limitations, addressing sources of potential bias, imprecision, generalisability, and, if relevant, multiplicity of analyses	20–21

## Appendix B. Examples of Post Topics for Which Educational Information Was Provided (Translated from Russian)

**Category**	**Description**	**Example Topics for Posts**
IBD	General information about IBD	1. What is IBD? 2. What is CD? 3. What is UC? 4. Who is at risk of IBD? 5. What is the prevalence of IBD?
Lifestyle	Adapting daily life with IBD	1. Planning downtime when you have IBD 2. IBD and exercise: what’s safe? 3. IBD and work: how to set up a comfortable environment 4. Traveling with IBD: how to prepare 5. IBD and alcohol: is it okay to drink?
Nutrition	Dietary advice for managing symptoms	1. IBD diet: the basics 2. What to eat (and what to avoid) during a flare 3. The low FODMAP diet (and recipes) 4. Packaged foods: what is IBD-friendly? 5. Eating well on trips and vacations
Treatment	Managing IBD with medication and care	1. Key IBD drugs: how to take them 2. How to stay on top of your meds 3. Biologics: what they are and who they are for 4. Managing side effects of treatment 5. Surgery for IBD: when is it needed?
Women’s health	How IBD affects women specifically	1. IBD and pregnancy: risks and planning 2. How IBD affects your period 3. Family planning with IBD 4. Hormone replacement therapy and IBD 5. IBD and menopause
Psychological support	Managing stress and mental well-being	1. Stress and IBD: breaking the cycle 2. Anxiety and depression in IBD 3. Body image in IBD 4. Talking to loved ones about your illness 5. Relaxation techniques for IBD 6. Support groups and therapy
Intimate life	How IBD affects relationships	1. Bringing up IBD with your partner 2. IBD and low libido: getting your confidence back 3. Practical tips for a comfortable sex life 4. Sexual dysfunction and IBD
Practical tips	Real-world advice for day-to-day management	1. How to spot a flare early 2. Your IBD go-bag: what to keep handy 3. Coping with IBD-related fatigue 4. Best apps for tracking your symptoms 5. Health gadgets that can help with IBD
CD—Crohn’s disease; FODMAP—Fermentable Oligo-, Di-, Mono-saccharides And Polyols; IBD—inflammatory bowel disease; UC—ulcerative colitis.		

## Appendix D

**Table A1:** The baseline characteristics of the recruited patients (Full Analysis Set population).

Parameter	*N*	Sample ^1^	Control Group ^1^	Telemonitoring Group ^1^	*p*-Value ^2^	BH-Adjusted *p*-Value ^3^
Demographic and clinical characteristics						
Sex	116				0.353	0.879
Male		60 (51.7%)	33 (56.9%)	27 (46.6%)		
Female		56 (48.3%)	25 (43.1%)	31 (53.4%)		
Age, years	116	34.5 [26.5; 42.0]	37.5 [28.0; 45.0]	32.0 [24.0; 38.0]	0.022	0.495
Type of IBD	116				0.999	0.999
UC		53 (45.7%)	27 (46.6%)	26 (44.8%)		
CD		63 (54.3%)	31 (53.4%)	32 (55.2%)		
Disease duration, years	116	4.0 [2.0; 9.0]	6.0 [2.0; 10.0]	3.0 [1.0; 7.0]	0.045	0.495
Clinical activity (HBI/SCCAI)	105				0.527	0.879
Remission		52 (49.5%)	26 (44.8%)	26 (55.3%)		
Mild activity		36 (34.3%)	20 (34.5%)	16 (34.0%)		
Moderate activity		12 (11.4%)	8 (13.8%)	4 (8.5%)		
Severe activity		5 (4.8%)	4 (6.9%)	1 (2.1%)		
Clinical activity (HBI/SCCAI), binary	105				0.119	0.514
Remission		52 (49.5%)	26 (44.8%)	26 (55.3%)		
Flare		51 (48.6%)	32 (55.2%)	19 (40.4%)		
Endoscopic disease activity	96				0.863	0.976
Remission		28 (29.2%)	16 (30.8%)	12 (27.3%)		
Mild activity		29 (30.2%)	17 (32.7%)	12 (27.3%)		
Moderate activity		27 (28.1%)	13 (25.0%)	14 (31.8%)		
Severe activity		12 (12.5%)	6 (11.5%)	6 (13.6%)		
Histological disease activity	86				0.607	0.886
No activity		20 (23.3%)	10 (20.4%)	10 (27.0%)		
Activity present		66 (76.7%)	39 (79.6%)	27 (73.0%)		
CT/MRE activity	49				0.541	0.879
No activity		16 (32.7%)	11 (36.7%)	5 (26.3%)		
Activity present		33 (67.3%)	19 (63.3%)	14 (73.7%)		
Patient-reported outcomes						
SIBDQ, score	104	48.5 [37.0; 58.0]	47.0 [37.0; 58.0]	49.5 [39.0; 59.0]	0.901	0.976
HRQoL (SIBDQ)	104				0.693	0.886
Suboptimal HRQoL		55 (52.9%)	32 (55.2%)	23 (50.0%)		
Optimal HRQoL		49 (47.1%)	26 (44.8%)	23 (50.0%)		
WHOQOL-26, physical health	103	0.6 [0.4; 0.7]	0.6 [0.4; 0.7]	0.6 [0.4; 0.7]	0.45	0.879
WHOQOL-26, psychological health	103	0.7 [0.6; 0.8]	0.7 [0.6; 0.7]	0.7 [0.6; 0.8]	0.219	0.713
WHOQOL-26, social relationships	103	0.8 [0.6; 0.8]	0.7 [0.5; 0.8]	0.8 [0.7; 0.8]	0.095	0.495
WHOQOL-26, environmental health	103	0.7 [0.6; 0.8]	0.7 [0.6; 0.8]	0.7 [0.6; 0.8]	0.537	0.879
GMAS, score	101	29.0 [26.0; 31.0]	29.5 [27.0; 31.0]	29.0 [25.0; 32.0]	0.536	0.879
Medication adherence (GMAS)	101				0.184	0.684
Non-adherent		28 (27.7%)	13 (22.4%)	15 (34.9%)		
Adherent		73 (72.3%)	45 (77.6%)	28 (65.1%)		
HADS-Depression, score	97	5.0 [3.0; 8.0]	5.0 [3.0; 8.0]	5.0 [3.0; 8.0]	0.751	0.887
Depression (HADS)	97				0.686	0.886
No depression		68 (70.1%)	37 (68.5%)	31 (72.1%)		
Subclinical depression		19 (19.6%)	10 (18.5%)	9 (20.9%)		
Clinically significant depression		10 (10.3%)	7 (13.0%)	3 (7.0%)		
HADS-Anxiety, score	97	7.0 [4.0; 10.0]	6.0 [4.0; 9.0]	7.0 [3.0; 11.0]	0.475	0.879
Anxiety (HADS)	97				0.275	0.793
No anxiety		55 (56.7%)	32 (59.3%)	23 (53.5%)		
Subclinical anxiety		24 (24.7%)	15 (27.8%)	9 (20.9%)		
Clinically significant anxiety		18 (18.6%)	7 (13.0%)	11 (25.6%)		
VSI, score	100	32.0 [22.0; 42.5]	32.0 [21.0; 43.0]	31.0 [22.0; 42.0]	0.716	0.886
Visceral sensitivity (VSI)	100				0.95	0.988
No visceral hypersensitivity		7 (7.0%)	4 (7.3%)	3 (6.7%)		
Moderate visceral hypersensitivity		40 (40.0%)	21 (38.2%)	19 (42.2%)		
Severe visceral hypersensitivity		53 (53.0%)	30 (54.5%)	23 (51.1%)		
TAS-26, score	98	66.0 [58.0; 71.0]	62.5 [57.0; 69.0]	68.0 [62.0; 71.0]	0.06	0.495
Alexithymia (TAS-26)	97				0.085	0.495
No alexithymia		40 (41.2%)	27 (50.9%)	13 (29.5%)		
Possible alexithymia		44 (45.4%)	21 (39.6%)	23 (52.3%)		
Clinically significant alexithymia		13 (13.4%)	5 (9.4%)	8 (18.2%)		
PSQ-18, score	86	77.0 [70.0; 84.0]	76.0 [70.0; 80.0]	78.0 [68.0; 84.0]	0.646	0.886

^1^ Continuous variables are presented as Median [Q1; Q3] (interquartile range, IQR), and categorical variables as *n* (%). ^2^ Fisher’s exact test; Wilcoxon rank sum test. ^3^ Benjamini & Hochberg correction for multiple testing. CD—Crohn’s disease; CT—computed tomography; GMAS—General Medication Adherence Scale; HADS—Hospital Anxiety and Depression Scale; HBI—Harvey–Bradshaw Index; HRQoL—Health-related quality of life; MRE—magnetic resonance enterography; PSQ-18—Patient Satisfaction Questionnaire; SCCAI—Simple Clinical Colitis Activity Index; SIBDQ—Short Inflammatory Bowel Disease Questionnaire; TAS-26—Toronto Alexithymia Scale; UC—ulcerative colitis; VSI—Visceral Sensitivity Index; WHOQOL-26—World Health Organization’s QoL.

**Table A2:** Comparative characteristics of patients in the telemonitoring group at baseline and after 6 months.

Parameter	*N*	Baseline ^1^	*N*	6 Months ^1^	*p*-Value ^2^	BH-Adjusted *p*-Value ^3^
Demographic and clinical characteristics						
Clinical activity (HBI/SCCAI)	32		32		0.024	0.060
Remission		19 (59.4%)		28 (87.5%)		
Mild activity		11 (34.4%)		4 (12.5%)		
Moderate activity		2 (6.3%)		0 (0.0%)		
Severe activity		0 (0.0%)		0 (0.0%)		
Endoscopic disease activity	26		12		0.392	0.522
Remission		9 (34.6%)		8 (66.7%)		
Mild activity		9 (34.6%)		2 (16.7%)		
Moderate activity		6 (23.1%)		2 (16.7%)		
Severe activity		2 (7.7%)		0 (0.0%)		
Histological disease activity	24		9		0.617	0.726
No activity		9 (37.5%)		6 (66.7%)		
Activity present		15 (62.5%)		3 (33.3%)		
Patient-reported outcomes						
SIBDQ, score	32	53.0 [41.0; 59.5]	32	57.0 [45.0; 63.5]	<0.001	0.004 *
HRQoL (SIBDQ)	32		32		0.221	0.368
Suboptimal HRQoL		14 (43.8%)		10 (31.3%)		
Optimal HRQoL		18 (56.3%)		22 (68.8%)		
WHOQOL-26, physical health	31	0.7 [0.5; 0.8]	32	0.7 [0.6; 0.8]	0.002	0.008 *
WHOQOL-26, psychological health	31	0.7 [0.6; 0.8]	32	0.8 [0.6; 0.8]	0.258	0.396
WHOQOL-26, social relationships	31	0.8 [0.7; 0.8]	32	0.7 [0.6; 0.8]	0.932	0.932
WHOQOL-26, environmental health	31	0.7 [0.6; 0.8]	32	0.8 [0.6; 0.8]	0.508	0.635
GMAS, score	29	29.0 [25.0; 32.0]	32	32.0 [30.0; 32.5]	<0.001	0.004 *
Medication adherence (GMAS)	29		31		0.023	0.060
Non-adherent		11 (37.9%)		4 (12.9%)		
Adherent		18 (62.1%)		27 (87.1%)		
HADS-Depression, score	31	5.0 [3.0; 7.0]	32	4.0 [2.0; 6.0]	0.151	0.302
Depression (HADS)	31		32		0.819	0.910
No depression		27 (87.1%)		26 (81.3%)		
Subclinical depression		3 (9.7%)		5 (15.6%)		
Clinically significant depression		1 (3.2%)		1 (3.1%)		
HADS-Anxiety, score	31	7.0 [2.0; 10.0]	32	5.5 [3.5; 8.5]	0.195	0.355
Anxiety (HADS)	31		32		0.314	0.449
No anxiety		19 (61.3%)		22 (68.8%)		
Subclinical anxiety		7 (22.6%)		6 (18.8%)		
Clinically significant anxiety		5 (16.1%)		4 (12.5%)		
VSI, score	31	29.0 [21.0; 40.0]	32	21.5 [15.5; 28.0]	<0.001	0.004 *
Visceral sensitivity (VSI)	31		32		0.007	0.027 *
No visceral hypersensitivity		3 (9.7%)		5 (15.6%)		
Moderate visceral hypersensitivity		15 (48.4%)		21 (65.6%)		
Severe visceral hypersensitivity		13 (41.9%)		6 (18.8%)		
TAS-26, score	31	68.0 [64.0; 71.0]	32	63.0 [55.0; 71.5]	0.018	0.060
Alexithymia (TAS-26)	31		32		0.062	0.138
No alexithymia		9 (29.0%)		15 (46.9%)		
Possible alexithymia		18 (58.1%)		11 (34.4%)		
Clinically significant alexithymia		4 (12.9%)	32	6 (18.8%)		
PSQ-18, score	31	78.0 [68.0; 84.0]	32	76.0 [67.5; 84.5]	0.922	0.932

^1^ Continuous variables are presented as Median [Q1; Q3] (interquartile range, IQR), and categorical variables as *n* (%). ^2^ McNemar’s test; Stuart–Maxwell test; Wilcoxon signed-rank test. ^3^ Benjamini & Hochberg correction for multiple testing. * *p* < 0.05. GMAS—General Medication Adherence Scale; HADS—Hospital Anxiety and Depression Scale; HBI—Harvey–Bradshaw Index; HRQoL—Health-related quality of life; PSQ-18—Patient Satisfaction Questionnaire; SCCAI—Simple Clinical Colitis Activity Index; SIBDQ—Short Inflammatory Bowel Disease Questionnaire; TAS-26—Toronto Alexithymia Scale; VSI—Visceral Sensitivity Index; WHOQOL-26—World Health Organization’s QoL.

**Table A3:** Comparative characteristics of patients in the control group at baseline and after 6 months.

Parameter	*N*	Baseline ^1^	*N*	6 Months ^1^	*p*-Value ^2^	BH-Adjusted *p*-Value ^3^
Demographic and clinical characteristics						
Clinical activity (HBI/SCCAI)	36		36		0.497	0.802
Remission		20 (55.6%)		24 (66.7%)		
Mild activity		12 (33.3%)		10 (27.8%)		
Moderate activity		4 (11.1%)		2 (5.6%)		
Severe activity		0 (0.0%)		0 (0.0%)		
Endoscopic disease activity	31		16		0.044	0.539
Remission		9 (29.0%)		6 (37.5%)		
Mild activity		12 (38.7%)		8 (50.0%)		
Moderate activity		8 (25.8%)		2 (12.5%)		
Severe activity		2 (6.5%)		0 (0.0%)		
Histological disease activity	30		11		0.48	0.802
No activity		6 (20.0%)		4 (36.4%)		
Activity present		24 (80.0%)		7 (63.6%)		
Patient-reported outcomes						
SIBDQ, score	36	51.5 [39.5; 59.5]	36	54.5 [45.5; 59.0]	0.163	0.696
HRQoL (SIBDQ)	36		36		0.182	0.696
Suboptimal HRQoL		17 (47.2%)		12 (33.3%)		
Optimal HRQoL		19 (52.8%)		24 (66.7%)		
WHOQOL-26, physical health	36	0.6 [0.5; 0.7]	33	0.6 [0.6; 0.8]	0.365	0.696
WHOQOL-26, psychological health	36	0.7 [0.6; 0.7]	33	0.6 [0.6; 0.8]	0.918	0.999
WHOQOL-26, social relationships	36	0.7 [0.5; 0.8]	33	0.8 [0.6; 0.8]	0.637	0.95
WHOQOL-26, environmental health	36	0.7 [0.6; 0.8]	33	0.7 [0.6; 0.8]	0.974	0.999
GMAS, score	36	0.6 [0.5; 0.7]	33	0.6 [0.6; 0.8]	0.365	0.696
Medication adherence (GMAS)	36	0.7 [0.6; 0.7]	33	0.6 [0.6; 0.8]	0.918	0.999
Non-adherent		7 (19.4%)		11 (37.9%)		
Adherent		29 (80.6%)		18 (62.1%)		
HADS-Depression, score	35	5.0 [3.0; 9.0]	34	6.0 [4.0; 8.0]	0.679	0.95
Depression (HADS)	35		34		0.076	0.539
No depression		23 (65.7%)		22 (64.7%)		
Subclinical depression		6 (17.1%)		11 (32.4%)		
Clinically significant depression		6 (17.1%)		1 (2.9%)		
HADS-Anxiety, score	35	7.0 [4.0; 10.0]	33	8.0 [4.0; 11.0]	0.234	0.696
Anxiety (HADS)	35		34		0.223	0.696
No anxiety		19 (54.3%)		15 (44.1%)		
Subclinical anxiety		11 (31.4%)		9 (26.5%)		
Clinically significant anxiety		5 (14.3%)		10 (29.4%)		
VSI, score	35	33.0 [22.0; 44.0]	34	30.0 [23.0; 41.0]	0.791	0.999
Visceral sensitivity (VSI)	35		34		0.344	0.696
No visceral hypersensitivity		2 (5.7%)		2 (5.9%)		
Moderate visceral hypersensitivity		12 (34.3%)		15 (44.1%)		
Severe visceral hypersensitivity		21 (60.0%)		17 (50.0%)		
TAS-26, score	34	62.5 [56.0; 69.0]	34	63.0 [56.0; 74.0]	0.877	0.999
Alexithymia (TAS-26)	34		34		0.077	0.539
No alexithymia		17 (50.0%)		16 (47.1%)		
Possible alexithymia		15 (44.1%)		10 (29.4%)		
Clinically significant alexithymia		2 (5.9%)		8 (23.5%)		
PSQ-18, score	22	78.0 [72.0; 87.0]	34	76.5 [69.0; 80.0]	0.277	0.696

^1^ Continuous variables are presented as Median [Q1; Q3] (interquartile range, IQR), and categorical variables as *n* (%). ^2^ McNemar’s test; Stuart–Maxwell test; Wilcoxon signed-rank test. ^3^ Benjamini & Hochberg correction for multiple testing. GMAS—General Medication Adherence Scale; HADS—Hospital Anxiety and Depression Scale; HBI—Harvey–Bradshaw Index; HRQoL—Health-related quality of life; PSQ-18—Patient Satisfaction Questionnaire; SCCAI—Simple Clinical Colitis Activity Index; SIBDQ—Short Inflammatory Bowel Disease Questionnaire; TAS-26—Toronto Alexithymia Scale; VSI—Visceral Sensitivity Index; WHOQOL-26—World Health Organization’s QoL.

## Appendix E

**Table A4:** Associations between telemonitoring and patient-reported outcomes at 6 months.

Outcome	β (Telemonitoring vs. Standard Care)	95% CI	*p*-Value
SIBDQ	3.44	−0.45–7.3	0.082
WHOQOL-26, physical health	0.06	0.00–0.11	0.050
WHOQOL-26, psychological health	0.02	−0.05–0.08	0.633
WHOQOL-26, social relationships	0.00	−0.10–0.10	0.980
WHOQOL26, environmental health	0.02	−0.04–0.08	0.454
HADS-Anxiety	−1.76	−3.24–−0.28	0.021 *
HADS-Depression	−1.06	−2.34–0.22	0.103
VSI	−5.08	−9.89–−0.26	0.039 *
GMAS	1.75	0.48–3.02	0.008 *
TAS-26	−3.07	−7.35–1.21	0.157
PSQ-18	1.16	−3.08–5.41	0.584

* *p* < 0.05. CI—confidence interval; GMAS—General Medication Adherence Scale; HADS—Hospital Anxiety and Depression Scale; PSQ-18—Patient Satisfaction Questionnaire; SIBDQ—Short Inflammatory Bowel Disease Questionnaire; TAS–26—Toronto Alexithymia Scale; VSI—Visceral Sensitivity Index; WHOQOL-26—World Health Organization’s QoL.

**Table A5:** Associations between telemonitoring and binary patient-reported outcomes at 6 months.

Outcome	OR	95% CI	*p*-Value
Optimal HRQoL (SIBDQ)	1.02	0.27–3.78	>0.90
Anxiety present	0.25	0.07–0.90	0.034 *
Depression present	0.49	0.13–1.93	0.311
Visceral hypersensitivity present	0.30	0.04–2.18	0.235
Adequate medication adherence	6.61	0.57–76.01	0.130
Alexithymia present	0.48	0.13–1.73	0.263
Clinical activity	3.57	0.99–12.91	0.053

* *p* < 0.05. CI—confidence interval; HRQoL—Health-related QoL; OR—odds ratio; SIBDQ—Short Inflammatory Bowel Disease Questionnaire.
